# Vasorelaxing Action of the Kynurenine Metabolite, Xanthurenic Acid: The Missing Link in Endotoxin-Induced Hypotension?

**DOI:** 10.3389/fphar.2017.00214

**Published:** 2017-05-01

**Authors:** Francesco Fazio, Albino Carrizzo, Luana Lionetto, Antonio Damato, Luca Capocci, Mariateresa Ambrosio, Giuseppe Battaglia, Valeria Bruno, Michele Madonna, Maurizio Simmaco, Ferdinando Nicoletti, Carmine Vecchione

**Affiliations:** ^1^Istituto Neurologico Mediterraneo (IRCCS),Pozzilli, Italy; ^2^Advanced Molecular Diagnostic, IDI Istituto Dermopatico dell’Immacolata (IRCCS),Rome, Italy; ^3^Department of Physiology and Pharmacology, Sapienza University of RomeRome, Italy; ^4^School of Medicine and Psychology, NESMOS Department, Sant’Andrea Hospital, Sapienza University of RomeRome, Italy; ^5^Department of Medicine and Surgery, University of SalernoBaronissi, Italy

**Keywords:** xanthurenic acid, vascular tone, lipopolysaccharide, hypotension, endotoxic shock

## Abstract

The *kynurenine pathway* of tryptophan metabolism is activated by pro-inflammatory cytokines. L-kynurenine, an upstream metabolite of the pathway, acts as a putative endothelium-derived relaxing factor, and has been hypothesized to play a causative role in the pathophysiology of inflammation-induced hypotension. Here, we show that xanthurenic acid (XA), the transamination product of 3-hydroxykynurenine, is more efficacious than L-kynurenine in causing relaxation of a resistance artery, but fails to relax pre-contracted aortic rings. In the mesenteric artery, XA enhanced activating phosphorylation of endothelial nitric oxide synthase (NOS), and the relaxing action of XA was abrogated by pharmacological inhibition of NOS and endothelial-derived hyperpolarizing factor. Systemic injection of XA reduced blood pressure in mice, and serum levels of XA increased by several fold in response to a pulse with the endotoxin, lipopolysaccharide (LPS). LPS-induced hypotension in mice was prevented by pre-treatment with the kynurenine monooxygenase (KMO) inhibitor, Ro-618048, which lowered serum levels of XA but enhanced serum levels of L-kynurenine. UPF 648, another KMO inhibitor, could also abrogate LPS-induced hypotension. Our data identify XA as a novel vasoactive compound and suggest that formation of XA is a key event in the pathophysiology of inflammation-induced hypotension.

## Introduction

**FIGURE 1:**
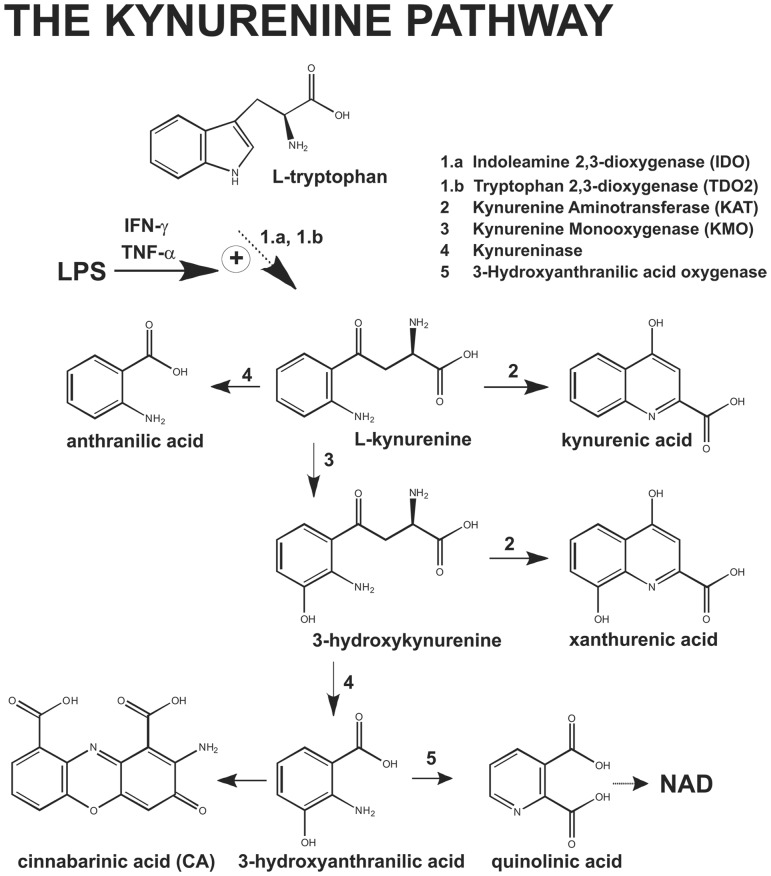
**Schematic representation of Kynurenine pathway**.

Hemodynamic shock and multiple organ failure are hallmarks of the systemic inflammatory response syndrome (SIRS), which may be caused by bacterial infections (Cohen, 2002). Lipopolysaccharide (LPS), which is released from the outer membrane of gram-negative bacteria lysed by immune cells (Raetz and Whitfield, 2002), plays a key role in the pathophysiology of endotoxic sepsis (Beutler and Rietschel, 2003). By interacting with toll-like receptor 4 (TLR4), LPS stimulates the production of tumor necrosis factor-α (TNF-α) (Carswell et al., 1975; Hoshino et al., 1999) and interferon-γ (IFN-γ, which in turn induce the enzyme indoleamine 2,3-dioxygenase (IDO) in a variety of cells, including immune and endothelial cells (Okuda et al., 1996; Wei et al., 2000; Mizutani et al., 2002; Jung et al., 2009; Wang et al., 2010). Tryptophan is metabolized by IDO or tryptophan 2,3-dioxygenase (TDO) into formylkynurenine, which is spontaneously converted into L-kynurenine (KYN). KYN in transaminated by kynurenine aminotransferases (KATs) into kynurenic acid (KYNA), a neuroactive compound that interacts with NMDA receptors and α_7_ nicotinic receptors. Alternatively, KYN is converted by kynurenine monooxygenase (KMO) into 3-hydroxykynurenine (3-HK), which can be transaminated into xanthurenic acid (XA) by KATs or, alternatively, metabolized into 3-hydroxyanthranylic acid (3-HANA) by kynureninase. 3-HANA is transformed into quinolinic acid (QUINA), which is further metabolized to produce nicotinamide (**Figure 1**).

A growing body of evidence suggests that metabolites generated within the kynurenine pathway are involved in the regulation of blood pressure. Polymorphic variants of the gene encoding for kynureninase are associated with changes in systolic blood pressure (SBP), and the transcript of kynureninase is about threefold higher in spontaneously hypertensive rats than in normotensive control rats (Mizutani et al., 2002). Regulation of vascular tone by kynurenine metabolites may become prominent during inflammation, i.e., under conditions in which the kynurenine pathway is strongly induced (Wang et al., 2010). Accordingly, pharmacological blockade of IDO protects mice against LPS-induced shock (Jung et al., 2009), and KYN has been shown to act as an endothelium-derived relaxing factor causing hypotension in mice treated with LPS or infected by Plasmodium *berghei* (Wang et al., 2010). Because the action of KYN on blood vessels was not mimicked by KYNA, 3-HK, 3-HANA or QUINA, it was concluded that KYN itself, rather than any of its metabolites, causes vascular relaxation under inflammatory conditions (Wang et al., 2010). However, the analysis of kynurenine metabolites did not include XA, which has long been considered as a mere detoxification product of 3-HK (Okuda et al., 1996; Wei et al., 2000). The recent demonstration that XA is biologically active, interacts with a not-yet identified G-protein-coupled receptor, and its plasma levels are largely reduced in patients affected by schizophrenia (Fazio et al., 2015) fueled interest in examining the role of XA in physiology and pathology. To our knowledge, the cardiovascular effects of XA have never been investigated with the exception of an old study showing that XA and other kynurenine metabolites cause bradycardia in isolated frog hearts (Rudzit et al., 1986).

We now report that XA displays a greater potency and efficacy than KYN in causing relaxation of a mouse resistance artery, and that its action required the presence of the endothelium and was mediated, at least in part, by an enhanced production of nitric oxide (NO·) and endothelium-derived hyperpolarizing factor (EDHF). More important, the hypothensive effect of LPS was abrogated by systemic treatment with selective inhibitors of KMO, the enzyme that converts KYN into the XA precursor, 3-HK.

## Materials and Methods

### Materials

Lipopolysaccharide (Salmonella enteriditis L6011), XA (4,8-dihydroxyquinoline-2-carboxylic acid), KYN sulfate, dansyl-norvaline (DNSnVal, internal standard for HPLC-MS/MS analysis), L-N^G^-nitroarginine methyl ester (L-NAME), apamin, and charybdotoxin (Ctx) were purchased from Sigma-Aldrich (St. Louis, MO, USA). 3,4-Dimethoxy-N-[4-(3-nitrophenyl) thiazol-2-yl] benzenesulfonamide (Ro-618048), (1S,2S)-2-(3,4-Dichlorobenzoyl) cyclopropanecarboxylic acid (UPF 648) and tetrodotoxin citrate were purchased from Tocris Cookson (Anawa Trading SA, Zurich, Switzerland; and Bristol, UK). Ro-618048 and UPF 648 were dissolved into sterile water adjusting the pH to 7.4 with 1N NaOH, and then diluted with sterile physiological solution to obtain a final concentration of 10 and 7.5 mg/mL, respectively. XA was dissolved into saline solution and the pH was adjusted to 7.4 with 1N NaOH. We did not use DMSO to avoid influences on vasocontraction (Pitts et al., 1986).

### Animals

All experiments involving animals were conform to the guidelines for the Care and Use of Laboratory Animals published from Directive 2010/63/EU of the European Parliament and were approved by IRCCS Neuromed review board.

Male adult C57Black/6N mice (22–24 g, body weight) were purchased from Charles River (Calco, Italy). Mice lacking type-2 metabotropic glutamate receptors (mGlu2 receptors) were originally provided by Prof. Shiegetada Nakanishi (Kyoto University, Japan). Mice were kept under environmentally controlled conditions (ambient temperature, 22°C; humidity, 40%) on a 12 h light/dark cycle with food and water *ad libitum.* Experiments were performed following the Guidelines for Animal Care and Use of the National Institutes of Health.

### Vascular Reactivity Studies

Segments of second-order of the superior mesenteric arteries were dissected free of fat and connective tissue in ice-cold Krebs solution, maintained at 4°C, and gassed with 95% O_2_ and 5% CO_2_. Studies of vascular reactivity on mouse mesenteric arteries were carried out as described previously (Vecchione et al., 2002; Carrizzo et al., 2016). In brief, mesenteric arteries were mounted on a wire myograph in organ chambers with Krebs solution and increasing concentrations of phenylephrine (10^-9^ to 10^-6^ M) were added to obtain a similar level of precontraction in each ring (80% of initial KCl-induced contraction). Caution was taken to avoid endothelium damage, and functional integrity of the endothelium was confirmed by the vasorelaxing response to acetylcholine (10^-9^ to 10^-6^ M). Vasorelaxation was expressed as per cent reduction of phenylephrine-induced contraction. Some experiments have been performed in endothelium-denuded vessels, as demonstrated by the absence of acetylcholine-induced vasorelaxation.

### Blood Pressure Measurements

Blood pressure was measured using the BP-2000 instrument (Visitech systems). The tail cuff method was carried out as described previously (Carrizzo et al., 2016). Blood pressure was measured in conscious mice treated with XA or its vehicle, or in mice treated with LPS (or its vehicle). In separate experiments, mice were challenged with LPS after pretreatment with Ro-618048 (40 mg/kg), UPF 648 (30 mg/kg), or their respective vehicle.

### Detection of Serum XA and KYN Levels by High Performance Liquid Chromatography/Tandem Mass Spectrometry (HPLC-MS/MS)

Serum levels of XA were measured in mice after the injection of XA (100 mg/kg) to obtain a pharmacokinetic profile of XA (time 0, 1, 2, 4, 8, and 24 h after i.p injection). Serum levels of XA and KYN were also measured in the blood collected at different times after LPS injection, or alternatively, at 24 h after injections of LPS, Ro-618048, or their combination. In these experiments, mice were anesthetized by isoflurane and blood was drawn from vena cava by a syringe at time 0, and then 2, 4, 24, and 36 h after LPS injection. Blood was allowed to stand at room temperature in a closed container for 30 min and then centrifuged for 1 min at 1000 relative centrifugal force (RCF). After centrifugation at 1500 ×*g* for 10 min, aliquots of serum were stored at -80°C until analysis. Serum levels of XA and KYN were measure by HPLC-MS/MS as detailed in ref. 11. In brief, HPLC analysis was performed using an Agilent Liquid Chromatography System series 1100 (Agilent Technologies, USA) a pentafluorophenyl column (100 mm × 2.1 mm, Kinetex PFP, 2.6 μm, 100 Å pore size, Phenomenex, Torrance, CA, USA) The mobile phase consisted of a solution of 0.1% aqueous formic acid (eluent A) and 100% of methanol (eluent B); elution was performed at flow rate of 300 μl/min by using an elution gradient. Mass spectrometry was performed on a 3200 triple quadrupole system (Applied Biosystems, Foster City, CA, USA) equipped with a Turbo Ion Spray source, as described previously (Fazio et al., 2015).

## Results

### Xanthurenic Acid Caused a Robust Relaxation of Mesenteric Arteries

**FIGURE 2:**
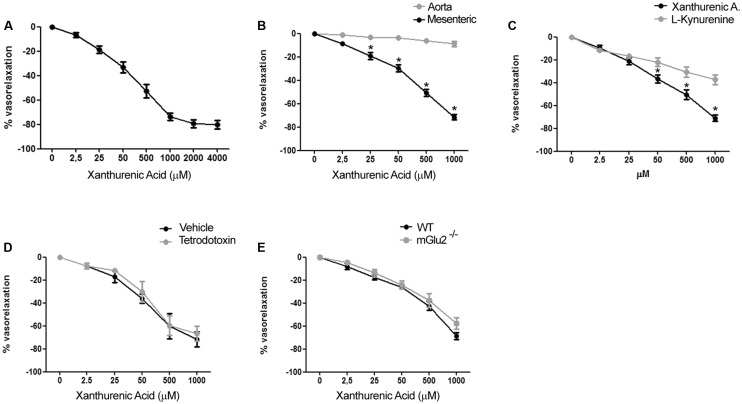
**Xanthurenic acid (XA) causes relaxation of isolated mouse mesenteric arteries. (A)** Concentration-response curve of XA-induced relaxation of phenylephrine-precontracted mesenteric artery. Values are means + S.E.M. of 6 determinations. **(B)** Vascular response of phenylephrine-precontracted mouse aorta rings or mesenteric arteries to increasing concentrations of XA (0–1000 μM). Values are means ± S.E.M. of eight determinations. ^∗^*p* < 0.05, (Two-way ANOVA + Bonferroni post-test). **(C)** Comparison of vascular response of phenylephrine-precontracted mouse mesenteric arteries to increasing concentrations of XA (0–1000 μM) or L-kynurenine (0–1000 μM). Values are means ± S.E.M. of six determinations. ^∗^*p* < 0.05, (Two-way ANOVA + Bonferroni post-test). **(D)** Vascular response of phenylephrine-precontracted mouse mesenteric arteries to increasing doses of XA in presence of (1 μM) tetrodotoxin. Values are means ± S.E.M. of 7 determination. **(E)** Vasorelaxing effect of XA in mesenteric arteries isolated from wild-type (WT) or mGlu2^-/-^ mice. Values are means ± S.E.M. of five determinations.

To examine the vascular action of XA, we performed experiments on isolated mouse mesenteric artery and aorta. Addition of XA caused a substantial relaxation of the mesenteric artery pre-contracted by the α_1_-adrenergic receptor agonist, phenylephrine. The action of XA was concentration-dependent with an apparent EC_50_ value of 70–80 μM and a plateau effect at 2 mM (**Figure 2A**). All further *in vitro* experiments were performed using XA concentration in the range from 2.5 μM to 1 mM. XA did not exert any vasorelaxant effect on mouse aortic rings (**Figure 2B**), suggesting that its action was specific for resistance vessels.

Because KYN is known to relax porcine coronary arteries (Wang et al., 2010), we compared the effects of XA and KYN in our mesenteric artery preparations. Remarkably, XA was about twofold as efficacious as KYN in causing vasorelaxation. At the highest concentrations tested (1 mM), XA and KYN caused relaxation of mesenteric arteries by 70–75% and <40%, respectively (**Figure 2C**). To examine whether the effects of XA were secondary to the release of vasoactive neurotransmitters or neuropeptides from perivascular nerve endings, we performed experiments in the presence of tetrodotoxin (1 μM), which prevents depolarization of presynaptic terminals by inhibiting voltage-sensitive sodium channels (Westcott and Segal, 2013). XA was equally potent and efficacious in relaxing mesenteric artery in the presence of tetrodotoxin (**Figure 2D**), suggesting that XA has a direct role on vascular function. XA was shown to activate mGlu2 and mGlu3 metabotropic glutamate receptors in heterologous expression systems, and some behavioral effects of XA in mice required the presence mGlu2 receptors (Fazio et al., 2015). The vasorelaxing action of XA was intact in mesenteric arteries isolated from mGlu2^-/-^ mice (**Figure 2E**), and we were unable to detect the transcripts of mGlu2 receptors in extracts from mesenteric arteries (not shown).

### Vasorelaxation Caused by Xanthurenic Acid Required the Presence of the Endothelium and Was Mediated by Nitric Oxide and Endothelium-derived Hyperpolarizing Factor

**FIGURE 3:**
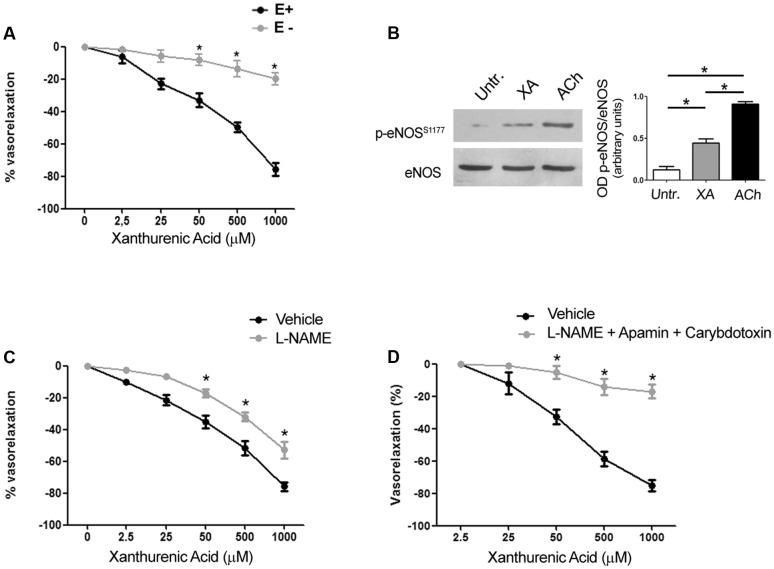
**Arterial relaxation induced by xanthurenic acid (XA) is mediated by nitric oxide and EDHF. (A)** Vascular response of phenylephrine-precontracted mouse mesenteric arteries to increasing concentrations of XA (0–1000 μM) in the presence (E+) or absence of endothelium (E–). Values are means ± S.E.M. of five determinations. ^∗^*p* < 0.05; (Two-way ANOVA + Bonferroni post-test) vs. E+. **(B)** Representative cropped immunoblot of mouse mesenteric arteries treated with 500 μM XA or 1 μM acetylcholine (ACh) used as positive control. Densitometric values are means ± S.E.M. of four determinations. ^∗^*p* < 0.05 (One-way ANOVA + Kruskal-Wallis test). **(C)** Vascular response of phenylephrine-precontracted mouse mesenteric arteries to increasing concentrations of XA (0–1000 μM) in the absence or presence of (300 μM) L-NAME. Values are means ± S.E.M. of six determinations. ^∗^*p* < 0.05: (Two-way ANOVA + Bonferroni post-test). **(D)** Vascular response of phenylephrine-precontracted mouse mesenteric arteries to increasing concentrations of XA (0–1000 μM) in presence of (300 μM) L-NAME plus a cocktail of EDHF inhibitors (50 nM apamin and 50 nM charybdotoxin). Values are means ± S.E.M. of six determinations. ^∗^*p* < 0.05 (Two-way ANOVA + Bonferroni post-test).

We performed experiments in endothelium-denuded mesenteric arteries, which failed to respond to acetylcholine-induced relaxation. The vasorelaxing action of XA was nearly abolished in the absence of endothelium (**Figure 3A**). In intact mesenteric arteries, XA enhanced activating serine 1177 phosphorylation of eNOS, as detected by immunoblot analysis (**Figure 3B**). In addition, the NOS pseudosubstrate, L-NAME (300 μM), partially reduced the vasorelaxing action of XA (**Figure 3C**). The action of XA was abrogated when L-NAME was combined with apamin (50 nM) and Ctx (50 nM), which selectively inhibit the EDHF pathway (Doughty et al., 1999; **Figure 3D**). These data demonstrate that both NO and EDHF are involved in the vasorelaxing action of XA.

### Xanthurenic Acid Lowered Blood Pressure in Mice and Increases in Serum Xanthurenic Acid Levels were Associated with LPS-induced Hypotension

**FIGURE 4:**
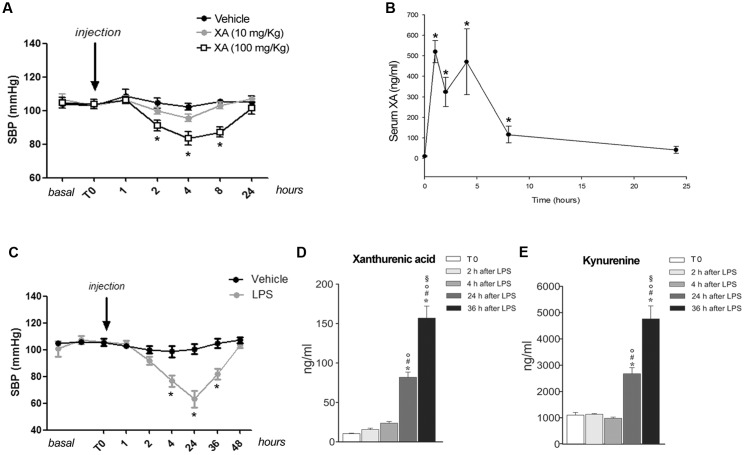
**Xanthurenic acid (XA) lowers blood pressure in mice and its serum levels are increased after injection of LPS. (A)** Systolic blood pressure (SBP) in C57BL/6 mice treated with vehicle or increasing doses of XA (10, 100 mg/kg). Values are means ± S.E.M. of 10–13 determinations. ^∗^*p* < 0.05; (One-way ANOVA + Bonferroni’s Multiple Comparison Test). **(B)** Serum levels of XA after the i.p. injection of XA (100 mg/kg); ^∗^*p* < 0.05 vs. time of XA injection (One-Way ANOVA + Fisher’s LSD). **(C)** SBP in mice treated with vehicle or LPS (1 mg/kg). Values are means ± S.E.M. of eight determinations. ^∗^*p* < 0.05; (Two-way ANOVA + Bonferroni’s Multiple Comparison Test). **(D,E)** Serum levels of XA and L-kynurenine at different time points after LPS injection. Blood samples were collected at different time points after LPS injection in anesthetized mice. Time 0 = 2 min prior LPS injection. Values are means ± S.E.M. of 3–4 determinations. ^∗^*p* < 0.05 vs. time of LPS injection; ^#^*p* < 0.05 vs. 2 h after LPS; °*p* < 0.05 vs. 4 h after LPS; ^x^ *p* < 0.05 vs. 24 after LPS (One-way ANOVA + Fisher’s LSD).

Knowing that resistance vessels are critically involved in blood pressure homeostasis we measured blood pressure in conscious mice at baseline and at 1, 2, 4, 8, and 24 h following i.p. injections of XA (10 or 100 mg/kg) or vehicle. XA treatment caused a clear-cut hypotensive effect that was already significant at 2 h, reached a plateau at 4–8 h, and disappeared at 24 h (**Figure 4A**). In a separate experiment, XA (100 mg/kg) was injected i.p., different groups of animals were killed before (T_0_), and 1, 2, 4, 8, and 24 h after injection for measurements of serum XA levels. As shown in **Figure 4B**, XA levels raised by about 50-fold at 1–4 h after injection, were still higher than 100 ng/ml at 8 h, and fell below 50 ng/ml at 24 h. We then measured blood pressure in response to a systemic pulse with LPS (1 mg/kg) in conscious mice, and, in a parallel experiment, we monitored serum levels of XA and KYN at corresponding time points in anesthetized mice. As expected, LPS caused a robust drop in blood pressure, which was already manifest at 4 h, peaked at 24 h, was still present at 36 h, and disappeared at 48 h (**Figure 4C**). LPS markedly enhanced both XA and KYN serum levels, mainly at the time that corresponded to the maximal hypotensive effect of LPS (24 h) (**Figures 4D,E**).

### Kynurenine Metabolism Downstream of KMO Was Required for the Hypotensive Effect of LPS

**FIGURE 5:**
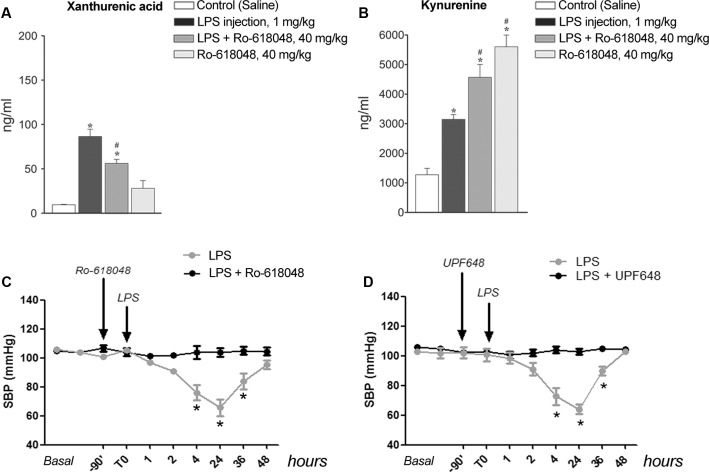
**Lipopolysaccharide-induced hypotension is prevented by pharmacological blockade of kynurenine monooxygenase. (A,B)** Serum levels of xanthurenic acid (XA) and L-kynurenine in mice injected i.p. with LPS (1 mg/kg) or vehicle 90 min after a pre-treatment with Ro-618048 (40 mg/kg) or its vehicle. Blood was collected from anesthetized mice 24 h after LPS injection. Values are means ± S.E.M. of three to four determinations. ^∗^*p* < 0.05 vs. control group (saline injection); ^#^*p* < 0.05 vs. LPS; (One-way ANOVA + Fisher’s LSD). **(C,D)** SBP in conscious mice challenged with LPS (1 mg/kg, i.p.) after pretreatment with Ro-618048, UPF 648 or their relative vehicle. Values are means + S.E.M. of eight determinations. ^∗^*p* < 0.05 (Two-way ANOVA + Bonferroni’s Multiple Comparison Test).

To examine whether formation of XA was a necessary requirement for LPS-induced hypotension, we performed *in vivo* experiments by treating mice with the KMO inhibitor, Ro-618048 (40 mg/kg, i.p.) (Moroni et al., 2005). KMO is a key enzyme of the kynurenine pathway that catalyzes the conversion of KYN into 3-HK, the direct precursor of XA. We could not adopt a more direct strategy because of the lack of drugs that *selectively* inhibit transamination of 3-HK into XA. Anesthetized mice were pre-treated with Ro-618048 or its vehicle 90 min prior to LPS or its vehicle, and blood was collected 24 h later for measurements of serum XA and KYN levels. In mice challenged with LPS, pretreatment with Ro-618048 reduced XA levels by about 35%. Ro-618048 had no effect on XA levels in the absence of LPS (**Figure 5A**). In contrast, Ro-618048 largely increased serum KYN levels both in the absence and in the presence of LPS (**Figure 5B**). We then examined in conscious mice whether the hypotensive response to LPS was affected by KMO inhibition with Ro-618048. Interestingly, pretreatment with Ro-618048 abolished the response to LPS at all time points up to 48 h (**Figure 5C**). To exclude that inhibition of LPS-induced hypotension by Ro-618048 was due to an off-target effect of the drug repeated the experiment using UPF 648, which behaves as a potent and selective KMO inhibitor, and is structurally unrelated to Ro-618048 (Ceresoli-Borroni et al., 2007). A 90-min pretreatment with UPF 648 (30 mg/kg, i.p.) also prevented LPS-induced hypotension (**Figure 5D**), suggesting that inflammation-induced hypotension requires the transformation of L-kynurenine into 3-hydroxykynurenine, with ensuing formation of XA.

### Statistical Analyses

Values of vascular reactivity were analyzed by Two-way ANOVA + Bonferroni’s *t*-test to isolate the differences. Densitometric values of immunoblots were analyzed by One-way ANOVA + Kruskal–Wallis test. Blood pressure values were analyzed by one-way ANOVA + Bonferroni’s Multiple Comparison Test. XA levels were analyzed by One-way ANOVA + Fisher’s LSD.

## Discussion

The kynurenine pathway is activated in response to pro-inflammatory cytokines in various cell types, including endothelial cells (Wang et al., 2010). Wang and colleagues have shown that activation of IDO was required for inflammation-induced vasodilation, and that KYN was the only tested metabolite of the pathway that caused arterial relaxation. KYN was active *in vitro* at relatively high concentrations (≥300 μM), but large amounts of KYN can be produced in endothelial cells under inflammatory conditions (Wang et al., 2010). KYN acted directly on smooth muscle cells by enhancing both cAMP and cGMP formation, and its action persisted in the absence of the endothelium (Wang et al., 2010). Our data offer a complementary scenario suggesting that XA is a new player in mechanisms of inflammation-induced hypotension. As opposed to KYN (Wang et al., 2010), XA cannot be considered as a putative endothelium-derived relaxing factor, because the action of exogenous XA required the presence of an intact endothelium. In addition, exogenous XA enhanced activating phosphorylation of eNOS and its relaxing activity was blunted by pharmacological inhibition of NOS, and by apamine and Ctx, which block the action of EDHF (Doughty et al., 1999) by inhibiting SK Ca^2+^-activated K^+^ channels and BK, IK, and some voltage-dependent potassium channels, respectively. Because the vasorelaxing action of XA was not entirely blocked by apamin and Ctx, it cannot be excluded that additional types of potassium channels are involved. Of note, the vasorelaxing action of L-kynurenine appears to be mediated by activation of Kv7 voltage-sensitive potassium channels (Sakakibara et al., 2015), although, as opposed to XA, the action of L-kynurenine on blood vessels does not require the integrity of the endothelium (Wang et al., 2010; Sakakibara et al., 2015). It is possible that XA and KYN cause arterial relaxation acting on different types of vessels and *via* different mechanisms. Accordingly, KYN caused relaxation of porcine coronary arteries, aortic rings, and mouse abdominal aorta (Wang et al., 2010), whereas XA was more efficacious than KYN in relaxing mouse mesenteric artery, but it failed to relax mouse aorta. The selectivity of XA for mesenteric artery with respect to aortic rings might reflect the different endothelium-dependent relaxing mechanisms in the two vessels. For example, endothelium-dependent aortic relaxation is mainly mediated by NO production, whereas endothelium-dependent mesenteric artery relaxation involves additional mechanisms, such as prostanoids and EDHF (Runnie et al., 2004). The vascular bed-specificity of endothelium-mediated mechanisms of relaxation/contraction is further supported by studies examining the response of different types of vessels, including aorta and mesenteric arteries, to mineralocorticoids (Mueller et al., 2015) or insulin/insulin-like growth factor-I (Wu et al., 1994).

How precisely XA acts to activate eNOS and the EDHF in endothelial cells remains to be determined. Experiments with tetrodotoxin excluded that the action of XA was mediated by the release of a vasoactive molecule from perivascular nerve endings. Because the vasorelaxing action of XA was concentration-dependent and a saturating effect was visible at concentrations >2 mM, it is likely that XA binds to a specific and saturable recognition site localized on endothelial membranes. The identity of the endothelial XA receptor is unknown. Arterial relaxation was not mediated by mGlu2 metabotropic glutamate receptors, which are involved in some behavioral effects of XA (Fazio et al., 2015). XA binds to specific and saturable recognition sites and enhances GTP-γ-S binding in brain membranes (Taleb et al., 2012). The elucidation of the molecular mechanism underlying XA-induced arterial relaxation awaits the identification of the putative G-protein coupled receptor activated by XA. The evidence that XA was able to cause arterial relaxation raised the possibility that XA could lower blood pressure in living animals and mediate, at least in part, SIRS-induced hypotension. Exogenous administration of XA (100 mg/kg) significantly reduced blood pressure at 2–8 h, when the corresponding serum XA levels were higher than 100 ng/ml (corresponding to 487 nM). Blood pressure returned back to normal at 24 h, when serum XA levels fell below 50 ng/ml (**Figures 4A,B**).

Lipopolysaccharide injection in mice caused the expected drop in blood pressure, which peaked at 24 h and was no longer observed at 48 h. Several mechanisms have implicated in LPS-induced hypotension, involving proinflammatory cytokines, inducible NOS (iNOS), integrin beta-2, superoxide anions, and prostaglandins (Michie et al., 1988; Mukaida et al., 1996; Obermeier et al., 1999; Liu et al., 2003). IDO is expressed in the endothelial cells of experimental animals and humans (Wang et al., 2010; Changsirivathanathamrong et al., 2011), and IDO activity in resistance vessels correlates with the extent of hypotension in human septic shock (Changsirivathanathamrong et al., 2011). That activation of the kynurenine pathway plays a key role in septic shock is suggested by the evidence that inhibition of IDO with 1-methyl-tryptophan prevents the drop in blood pressure in mice injected with LPS (Wang et al., 2010). In mice challenged with LPS, levels of both KYN and XA increased by several fold after 24 h, the time that corresponds to the peak of LPS-induced hypotension. Levels of both compounds continued to increase in the following 24 h, when levels of blood pressure turned back to normal. This suggests that homeostatic mechanisms that control blood pressure overcome the hypotensive effect of kynurenines at 48 h following LPS injection. Interestingly, a substantial reduction of blood pressure was observed 4 h following LPS injection, when KYN levels were unchanged, and XA levels showed a trend to an increase but remained lower than 50 ng/ml. These relatively low levels of XA might reach the threshold to cause hypotension in the presence of other vasoactive molecules produced in response to LPS (compare the two experiments described in **Figures 4A–D**). Although serum concentrations of XA in mice challenged with LPS (about 0.5–0.75 μM at 24–48 h) were >20-fold lower than the minimal vasorelaxing concentrations *in vitro*, it is likely that much greater concentrations of XA are reached in blood vessels because of a local synthesis in the endothelium, where the kynurenine pathway is activated by IDO.

Perhaps the most relevant finding of our study was that treatment of mice with the KMO inhibitor, Ro-618048 (Moroni et al., 2005), abrogated the effect of LPS on blood pressure although it caused large increase in serum KYN levels. This evidence strongly suggests that, at least in our model, KYN metabolism downstream of KMO is a critical event in the pathogenesis of LPS-induced hypotension. It is unlikely that Ro-618048 had off-target effects on the regulation of blood pressure because the drug was inactive in the absence of LPS, i.e., when the kynurenine pathway was not activated. In principle, any kynurenine metabolite lying downstream of KMO could have contributed to the hypotensive effect of LPS. Knowing that 3-HK, 3-HANA and QUINA cause no arterial relaxation *in vitro* (Wang et al., 2010), it is likely that Ro-618048 prevented LPS-induced hypotension by reducing the synthesis of XA from the KYN metabolite, 3-HK. Of note, however, pretreatment with Ro-618048 reduced, but did not abolish, the increase in serum XA levels induced by LPS. This leaves opened the possibility that other kynurenine metabolites that lie downstream of KMO (perhaps different from 3-HK, 3-HANA and QUINA) are involved in inflammation-induced hypotension. This hypothesis warrants further investigation. We could not use KAT inhibitors (Jayawickrama et al., 2015) to directly inhibit XA synthesis from 3-HK because KAT also canalizes the formation of KYNA from KYN (Jayawickrama et al., 2015; see also **Figure 1**). The two expected effects of KAT inhibitors (shunt of the pathway toward the metabolism of KYN into of 3-HK and inhibition of 3-HK transamination into XA), and perhaps other off-target effects, may confound the study of LPS-induced hypotension.

## Conclusion

Our findings demonstrate that XA selectively relaxes resistance vessels, and suggest that induction of XA synthesis is critically involved in the pathogenesis of endotoxin-induced hypotension. This is highly novel in the kynurenine field because, so far, the study of cardiovascular effects has been limited to “conventional” kynurenines, such as KYN, KYNA, 3-HK, 3-HANA, and QUINA. Our findings encourage the use of KMO inhibitors, which limit the production of the XA precursor, 3-HK, in the treatment of endotoxic shock. Again, the potential use of KAT inhibitors is limited by the multiple action of KATs within the kynurenine pathway. The design of drugs that selectively inhibit transamination of 3-HK into XA with limited effects on transamination of KYN into KYNA (a very difficult task) may offer new powerful instruments for the study of the biological activity of XA. If ever available, these drugs are expected to be highly effective in the treatment of endotoxic shock without the potential adverse effects of IDO and KMO inhibitors, which are upstream inhibitors of the kynurenine pathway and may limit the homeostatic actions of kynurenines in the CNS and immune system (Schwarcz et al., 2012).

## Author Contributions

All the authors have contributed significantly to the submitted work. FF, AC, LL, AD, LC, MA, and MM designed and performed experiments, analyzed data and wrote the manuscript. GB, VB, MS, FN, and CV designed the experiments and analyzed data. CV and FN made critical revision of the manuscript.

## Conflict of Interest Statement

The authors declare that the research was conducted in the absence of any commercial or financial relationships that could be construed as a potential conflict of interest.

## References

[B1] Beutler B. Rietschel E. T. (2003). Innate immune sensing and its roots: the story of endotoxin. *Nat. Rev. Immunol.* 3 169–176. 10.1038/nri1004 12563300

[B2] Carrizzo A. Ambrosio M. Damato A. Madonna M. Storto M. Capocci L. (2016). *Morus alba* extract modulates blood pressure homeostasis through eNOS signaling. *Mol. Nutr. Food Res.* 60 2304–2311. 10.1002/mnfr.201600233 27234065

[B3] Carswell E. A. Old L. J. Kassel R. L. Green S. Fiore N. Williamson B. (1975). An endotoxin-induced serum factor that causes necrosis of tumors. *Proc. Natl. Acad. Sci. U.S.A.* 72 3666–3670. 10.1073/pnas.72.9.3666 1103152 PMC433057

[B4] Ceresoli-Borroni G. Guidetti P. Amori L. Pellicciari R. Schwarcz R. (2007). Perinatal kynurenine 3-hydroxylase inhibition in rodents: pathophysiological implications. *J. Neurosci. Res.* 85 845–854. 10.1002/jnr.21183 17279543

[B5] Changsirivathanathamrong D. Wang Y. Rajbhandari D. Maghzal G. J. Mak W. M. Woolfe C. (2011). Tryptophan metabolism to kynurenine is a potential novel contributor to hypotension in human sepsis. *Crit. Care Med.* 39 2678–2683. 10.1097/CCM.0b013e31822827f2 21765346

[B6] Cohen J. (2002). The immunopathogenesis of sepsis. *Nature* 420 885–891. 10.1038/nature01326 12490963

[B7] Doughty J. M. Plane F. Langton P. D. (1999). Charybdotoxin and apamin block EDHF in rat mesenteric artery if selectively applied to the endothelium. *Am. J. Physiol.* 276 H1107–H1112. 10070099 10.1152/ajpheart.1999.276.3.H1107

[B8] Fazio F. Lionetto L. Curto M. Iacovelli L. Cavallari M. Zappulla C. (2015). Xanthurenic acid activates mGlu2/3 metabotropic glutamate receptors and is a potential trait marker for schizophrenia. *Sci. Rep.* 5:17799 10.1038/srep17799 PMC4672300 26643205

[B9] Hoshino K. Takeuchi O. Kawai T. Sanjo H. Ogawa T. Takeda Y. (1999). Cutting edge: toll-like receptor 4 (TLR4)-deficient mice are hyporesponsive to lipopolysaccharide: evidence for TLR4 as the *Lps* gene product. *J. Immunol.* 162 3749–3752. 10201887

[B10] Jayawickrama G. S. Sadig R. R. Sun G. Nematollahi A. Nadvi N. A. Hanrahan J. R. (2015). Kynurenine aminotransferases and the prospects of inhibitors for the treatment of schizophrenia. *Curr. Med. Chem.* 22 2902–2918. 10.2174/0929867322666150608094054 26051411

[B11] Jung I. D. Lee M. G. Chang J. H. Lee J. S. Jeong Y. I. Lee C. M. (2009). Blockade of indoleamine 2,3-dioxygenase protects mice against lipopolysaccharide-induced endotoxin shock. *J. Immunol.* 182 3146–3154. 10.4049/jimmunol.0803104 19234212

[B12] Liu J. Batkai S. Pacher P. Harvey-White J. Wagner J. A. Cravatt B. F. (2003). Lipopolysaccharide induces anandamide synthesis in macrophages via CD14/MAPK/phosphoinositide 3-kinase/NF-kappaB independently of platelet-activating factor. *J. Biol. Chem.* 278 45034–45039. 10.1074/jbc.M306062200 12949078

[B13] Michie H. R. Manogue K. R. Spriggs D. R. Revhaug A. O’Dwyer S. Dinarello C. A. (1988). Detection of circulating tumor necrosis factor after endotoxin administration. *N. Engl. J. Med.* 318 1481–1486. 10.1056/NEJM198806093182301 2835680

[B14] Mizutani K. Sugimoto K. Okuda T. Katsuya T. Miyata T. Tanabe T. (2002). Kynureninase is a novel candidate gene for hypertension in spontaneously hypertensive rats. *Hypertens. Res.* 25 135–140. 10.1291/hypres.25.135 11924719

[B15] Moroni F. Cozzi A. Carpendo R. Cipriani G. Veneroni O. Izzo E. (2005). Kynurenine 3-mono-oxygenase inhibitors reduce glutamate concentration in the extracellular spaces of the basal ganglia but not in those of the cortex or hippocampus. *Neuropharmacology* 48 788–795. 10.1016/j.neuropharm.2004.10.019 15829251

[B16] Mueller K. B. Bender S. B. Hong K. Yang Y. Aronovitz M. Jaisser F. (2015). Endothelial mineralocorticoid receptors differentially contribute to coronary and mesenteric vascular function without modulating blood pressure. *Hypertension* 66 988–997. 10.1161/HYPERTENSIONAHA.115.06172 26351033 PMC4600033

[B17] Mukaida N. Ishikawa Y. Ikeda N. Fujioka N. Watanabe S. Kuno K. (1996). Novel insight into molecular mechanism of endotoxin shock: biochemical analysis of LPS receptor signaling in a cell-free system targeting NF-kappaB and regulation of cytokine production/action through beta2 integrin in vivo. *J. Leukoc. Biol.* 59 145–151. 8603986 10.1002/jlb.59.2.145

[B18] Obermeier F. Gross V. Scholmerich J. Falk W. (1999). Interleukin-1 production by mouse macrophages is regulated in a feedback fashion by nitric oxide. *J. Leukoc. Biol.* 66 829–836. 10577516 10.1002/jlb.66.5.829

[B19] Okuda S. Nishiyama N. Saito H. Katsuki H. (1996). Hydrogen peroxide-mediated neuronal cell death induced by an endogenous neurotoxin, 3-hydroxykynurenine. *Proc. Natl. Acad. Sci. U.S.A.* 93 12553–12558. 10.1073/pnas.93.22.12553 8901620 PMC38030

[B20] Pitts L. H. Young A. R. McCulloch J. MacKenzie E. (1986). Vasomotor effects of dimethyl sulfoxide on cat cerebral arteries in vitro and in vivo. *Stroke* 17 483–487. 10.1161/01.STR.17.3.483 3715947

[B21] Raetz C. R. Whitfield C. (2002). Lipopolysaccharide endotoxins. *Annu. Rev. Biochem.* 71 635–700. 10.1146/annurev.biochem.71.110601.135414 12045108 PMC2569852

[B22] Rudzit V. K. Silenietse G. O. Irgenson I. B. (1986). [Participation of kynurenine and its derivatives in disorders of the cardiac rhythm]. *Bull. Exp. Biol. Med.* 102 719–721. 3801625

[B23] Runnie I. Salleh M. N. Mohamed S. Head R. J. Abeywardena M. Y. (2004). Vasorelaxation induced by common edible tropical plant extracts in isolated rat aorta and mesenteric vascular bed. *J. Ethnopharmacol.* 92 311–316. 10.1016/j.jep.2004.03.019 15138017

[B24] Sakakibara K. Feng G. G. Li J. Akahori T. Yasuda Y. Nakamura E. (2015). Kynurenine causes vasodilation and hypotension induced by activation of KCNQ-encoded voltage-dependent K(+) channels. *J. Pharmacol. Sci.* 129 31–37. 10.1016/j.jphs.2015.07.042 26318674

[B25] Schwarcz R. Bruno J. P. Muchowski P. J. Wu H. Q. (2012). Kynurenines in the mammalian brain: when physiology meets pathology. *Nat. Rev. Neurosci.* 13 465–477. 10.1038/nrn3257 22678511 PMC3681811

[B26] Taleb O. Maammar M. Brumaru D. Bourguignon J. J. Schmitt M. Klein C. (2012). Xanthurenic acid binds to neuronal G-protein-coupled receptors that secondarily activate cationic channels in the cell line NCB-20. *PLoS ONE* 7:e48553 10.1371/journal.pone.0048553 PMC3491036 23139790

[B27] Vecchione C. Fratta L. Rizzoni D. Notte A. Poulet R. Porteri E. (2002). Cardiovascular influences of alpha1b-adrenergic receptor defect in mice. *Circulation* 105 1700–1707. 10.1161/01.CIR.0000012750.08480.55 11940550

[B28] Wang Y. Liu H. McKenzie G. Witting P. K. Stasch J. P. Hahn M. (2010). Kynurenine is an endothelium-derived relaxing factor produced during inflammation. *Nat. Med.* 16 279–285. 10.1038/nm.2092 20190767 PMC3556275

[B29] Wei H. Leeds P. Chen R. W. Wei W. Leng Y. Bredesen D. E. (2000). Neuronal apoptosis induced by pharmacological concentrations of 3-hydroxykynurenine: characterization and protection by dantrolene and Bcl-2 overexpression. *J. Neurochem.* 75 81–90. 10.1046/j.1471-4159.2000.0750081.x 10854250

[B30] Westcott E. B. Segal S. S. (2013). Ageing alters perivascular nerve function of mouse mesenteric arteries in vivo. *J. Physiol.* 591 1251–1263. 10.1113/jphysiol.2012.244483 23247111 PMC3607869

[B31] Wu H. Y. Jeng Y. Y. Yue C. J. Chyu K. Y. Hsueh W. A. Chan T. M. (1994). Endothelial-dependent vascular effects of insulin and insulin-like growth factor I in the perfused rat mesenteric artery and aortic ring. *Diabetes Metab. Res. Rev.* 43 1027–1032. 10.2337/diab.43.8.1027 8039596

